# Mechanical response and in-situ deformation mechanism of cortical bone materials under combined compression and torsion loads

**DOI:** 10.1371/journal.pone.0271301

**Published:** 2022-07-27

**Authors:** Xingdong Sun, Wandi Wu, Renbo Zhang, Hongru Qu, Jie Wang, Ke Xu, Liangfei Fang, Liangyuan Xu, Rui Jiang

**Affiliations:** School of Engineering, Anhui Agricultural University, Hefei, People’s Republic of China; University of Vigo, SPAIN

## Abstract

Bone fracture is an extremely dangerous health risk to human. Actually, cortical bone is often subjected to the complicated loading patterns. The mechanical properties and deformation mechanism under the complicated loading pattern could provide a more precise understanding for the bone fracture. For this purpose, the mechanical response and multi-scale deformation mechanism of cortical bone material were investigated by in-situ experimental research using the compression-torsion coupling loads as an example. It was found that the torsion strength and shear modulus all decreased under the compression-torsion coupling loads than single torsion load. This indicated bone would suffer greater risk of fracture under the compression-torsion coupling loads. Based on in-situ observation, it was found that the rapid reduction of the anisotropy of bone material under the compression load was the potential influencing factor. Because of the redistribution of the principal strain and the variations of cracks propagation, the comprehensive fracture pattern containing both transverse and longitudinal fracture was shown under the coupling loads, and finally resulted in the reduction of the torsion properties. This research could provide new references for researches on mechanical properties of cortical bone material under complicated loading patterns.

## Introduction

Cortical bone is a natural hierarchical biomaterial, and its multi-scale structure is crucial to the mechanical properties [1–7]. Cortical bone plays an important role in load passing, load bearing and organs protecting [5]. Bone fracture is the most intuitive failure behavior to the external loading. Investigating the mechanical properties could reveal the potential deformation mechanism of cortical bone, offering the theoretical basis for the researches on bone fractures. Torsion is a typical loading pattern, which could result in a serious bone fracture. The potential deformation mechanism of cortical bone under torsion load remains unclear. Thus, based on the objective importance of the behavior during fractures, the mechanical responses of cortical bone material under torsion load were broadly studied [8–10].

Jepsen explored the damage of bone under the loading-unloading cycle and the influence of the damage on the elastic modulus, yield strength and fracture characteristics. The result indicated that there was an important amplitude effect of degeneration on torsion properties [11]. The mechanical properties in the post-yield failure stage of the human femoral bone was investigated by Jepsen, and it was found that the cracks at the interface of the bone plates were the major factor to the redistribution of the internal shear stress in the post yield stage. The independent cracks delayed the final fracture [12]. In terms of investigating the relationship between the mechanical property and the structure of cortical bone, Guo found a great negative correlation between the longitudinal elastic modulus and the porosity under the torsion load [13]. Forwood found that the torque, average stress, stiffness and energy absorption of cortical bone decreased gradually with the increasing of loading cycle, and the number of the micro-cracks increased with the loading cycle [14].

As shown above, the researches on mechanical properties of cortical bone mostly concentrated on single torsion pattern. In reality, the loading pattern of cortical bone is complicated, usually containing more than one load. The deformation mechanisms of cortical bone under the coupling loading patterns were different from that under single loading condition [15,16]. Nand thought the paediatirc injuries were caused by the torsion or bending stresses under the pre-compressive stress due to the weight [17]. Previous investigations have also provided evidences that multi-axial loading affected the mechanical property of bone materials, with the potential mechanisms unknown [18–21]. Meanwhile, Jakob believed the study of mechanical properties of biological hard tissue materials would extend to the directions of lamella level, physiological environmental coupling and complex loading mode [22]. Willie thought that the research on mechanical properties of cortical bone under the objective complex loading pattern was beneficial to the precise design of structure of bone substitute [23]. Zainab considered that the treatment strategy on bone fracture was mainly based on clinician’s subjective judgment, but the evidence based information on bone strength and deformation mechanism under complicated loading patterns was absented [24].

In this paper, to understand the objective mechanical properties, the mechanical responses and the potential deformation mechanism of cortical bone under compression-torsion coupling loads were investigated by in-situ experimental research in multi-scale. This research would provide new perspectives to researches on mechanical property of cortical bone under the complicated loading patterns, and would provide references for researches on failure mechanism of cortical bone under close-to-service conditions.

## Materials and methods

### Bone specimens

**Fig 1:**
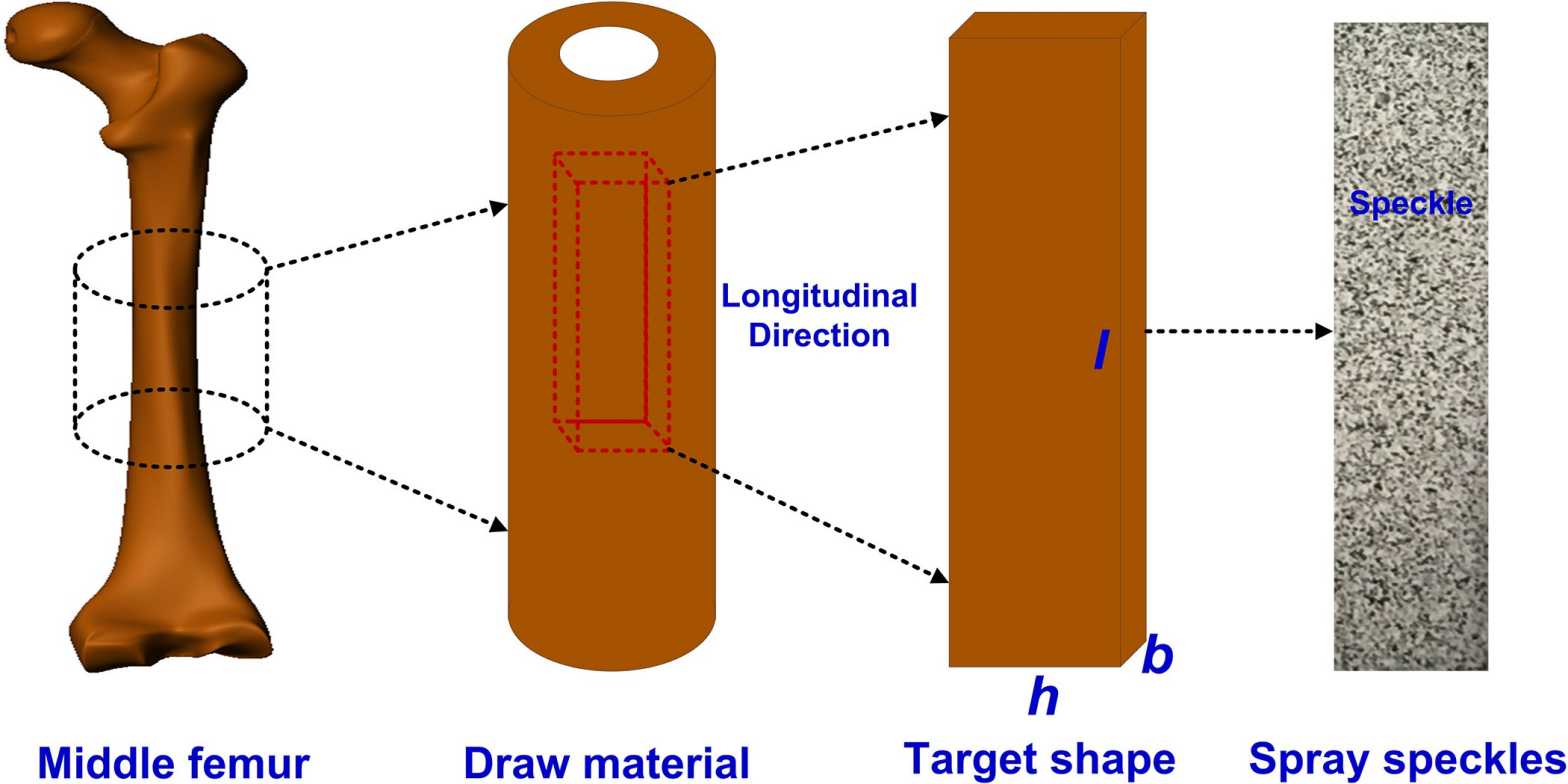
Scheme of obtainment process of cortical bone specimen.

Cortical bone specimens were obtained from middle femur of a pig being 1.5 years old, collected from Charoen Pokphand Group in Changchun city of Northeast of China. As shown in Fig 1, a rectangle block was obtained from the middle femur, and the specimens were cut along longitudinal direction with a band saw. In the cutting process, the phosphate buffered saline (PBS) was irrigated on the incision [25]. The rectangle block specimens were polished by using emery papers (P1000, P2000, P3000) step-by-step as coarse grinding. Then the precision grinding was carried out with diamond polishing solution. The final shape and physical dimension were shown in Fig 1 and Table 1, respectively. At last, the specimens were cleaned by the ultrasonic cleaner (KQ-218, SHUMEI, CHINA) for one hour. Until be used, the specimens were preserved by immersing in PBS under -20°C.

**Table 1:** The dimensions of the cortical bone specimen.

Material	Length(*h*)	Width(*b*)	Height(*l*)
**Cortical bone**	10.00mm	3.00mm	50.00mm

As shown in Fig 1, to investigate the evolution mechanism of the surface strain of cortical bone, the speckle with obvious contrast was sprayed on the specimens.

### Methods and instrument

In this research, for the purpose of obtaining the evolution of mechanical properties of cortical bone under compression-torsion coupling loads, the contrast experiments were designed. The experimental group and control group were the compression-torsion coupling loads and the single torsion load, respectively. The compression loading rate was set as 0.01mm/s, and the torsion loading rate was set as 0.3°/s. In the experiments, the coupling load was achieved by compression loading at first and torsion loading subsequently. To simulate the different compression conditions, the compression load was set as 500N, 1000N, 1500N, respectively. For each loading type, concluding single torsion loading type or compression-torsion coupling loading type with different compression load, the number of the bone specimens is 9. To investigate the effects of torsion loading speed to the torsion strength, a series of torsion loading speeds were set as 0.1°/s, 0.3°/s, 0.5°/s, 1°/s, 2°/s, 3°/s in the subsequent tests.

**Fig 2:**
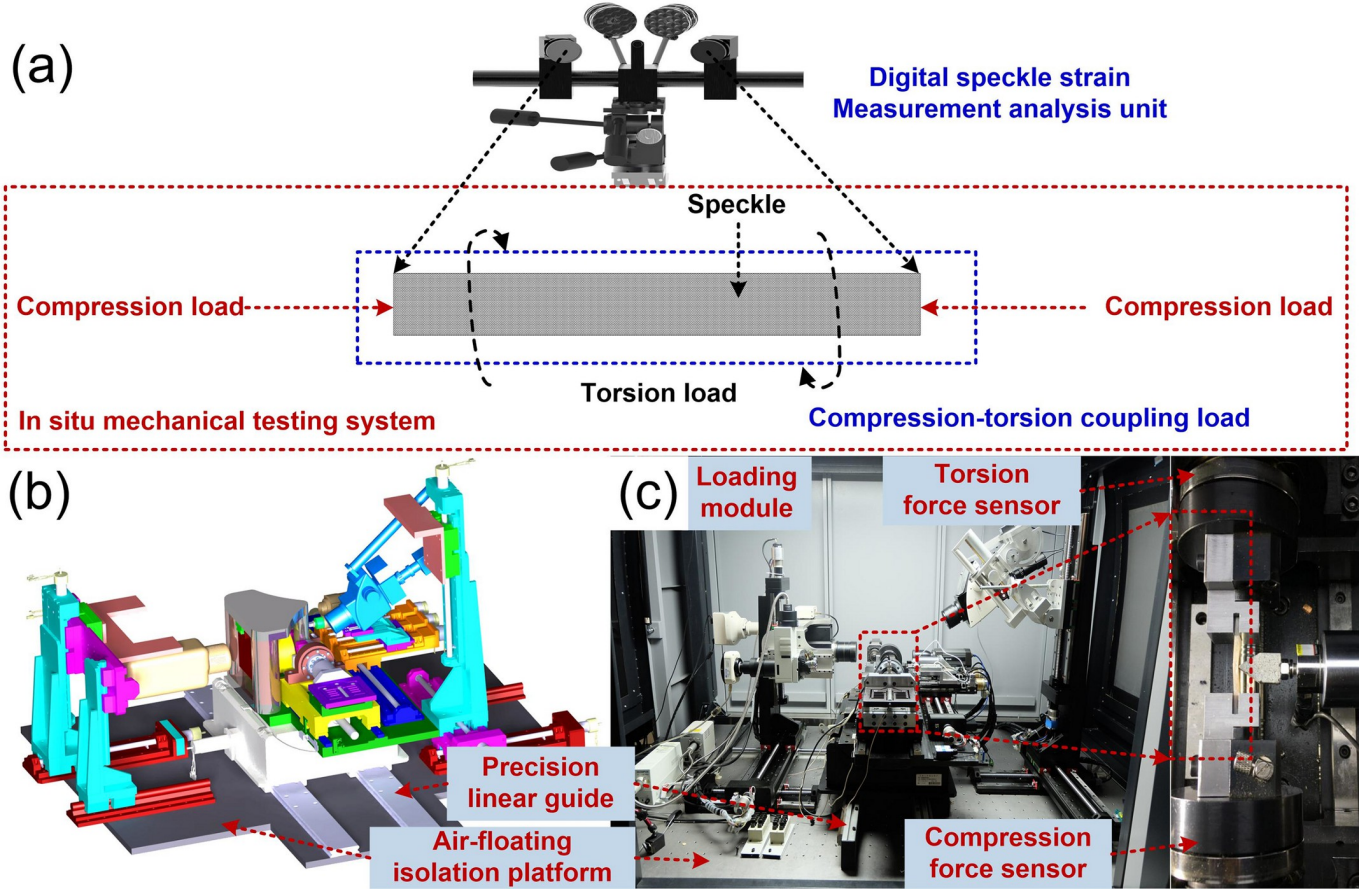
In-situ testing system of mechanical property of materials under multiple load patterns.

As shown in Fig 2, the experimental system was developed by our research group, including loading module, signal collecting module, in-situ observation module and air-floating isolation platform. The loading mode involves tension/compression load, torsion load, and compression-torsion coupling load pattern. The signal collecting module involves load sensor and displacement sensor, which was used to obtain the precise load and displacement during the experiment. The in-situ observation module involves the digital speckle strain measurement analysis unit, which can acquire the dynamic evolution mechanism of surface strain in real-time. For the purpose of ensuring the measurement accuracy, the experiments were carried out on the air-floating isolation platform.

The shear modulus is the ability of materials to resist the shear strain, and torsion strength is the ability of materials to resist the external torque. In this research, the shear modulus and torsion strength were selected to be characterized in order to describe the changing of the mechanical properties of cortical bone material under both single torsion load and compression-torsion coupling loads.

The shear modulus and the torsion strength could be calculated from Eqs (1)–(4).


$$G=\frac{\tau }{\gamma }=\frac{2TL_{0}}{\alpha b^{2}h\sqrt{b^{2}+h^{2}}\varphi }$$ (1)

$$\tau_{b}=\frac{T_{b}}{W_{t}}=\frac{T_{b}}{\alpha b^{2}h}$$ (2)

$$\tau =\frac{T}{W_{t}}$$ (3)

$$\gamma =\frac{\varphi d_{0}}{2L_{0}}=\frac{\varphi \sqrt{b^{2}+h^{2}}}{2L_{0}}$$ (4)

In the equations, *G*, *τ*_*b*_, *T*_*b*_, *τ*, *γ*, *h*, *b*, *L*_*0*_, *φ*, *T* are the shear modulus, torsion strength, maximum torque, shear stress, shear strain, width, thickness, gauge length of bone specimen, torsion angle, and torque, respectively.

*W*_*t*_ is the torsion section modulus of solid rectangle specimen, could be calculated from Eq (5).


$$W_{t}=\alpha b^{2}h$$ (5)

In the equation, *α* is the coefficient related to the ratio *h/b*. In this experiments, *h* = 10.0mm, *b* = 3.0mm. Based on Table 2, *α* = 0.272.

**Table 2:** The coefficient of the rectangle specimen under torsion load.

*h/b*	1.0	1.2	1.5	2.0	2.5	3.0	4.0	6.0	8.0	10.0	∞
***α***	0.208	0.219	0.231	0.246	0.258	0.267	0.282	0.299	0.307	0.313	0.333

## Results

**Fig 3:**
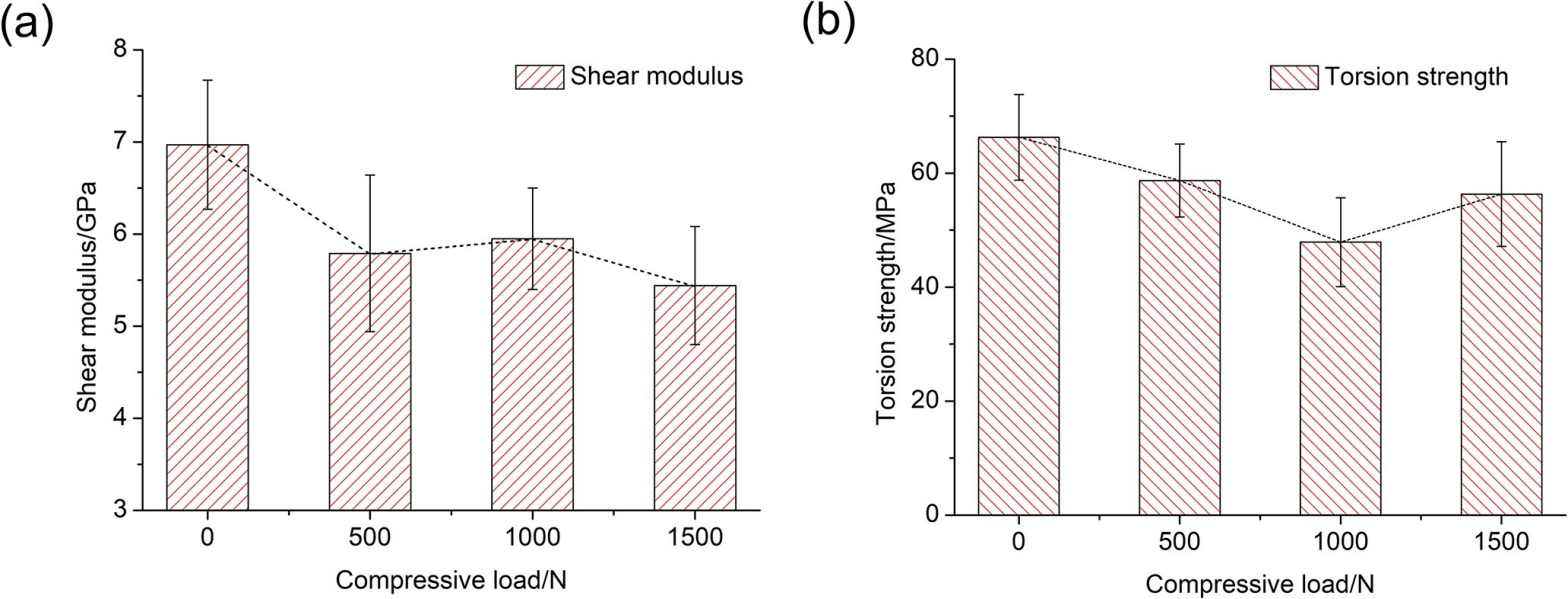
Variations of shear modulus and torsion strength with different compression load.

As shown in Fig 3, the variations of the shear modulus and the torsion strength under both the single torsion load and the compression-torsion coupling loads were obtained. Based on our calculation, when the compression load was 500N, the shear modulus and torsion strength decreased by 16.9 percent and 11.5 percent, respectively. When the compression load was 1000N, the shear modulus and torsion strength decreased by 14.6 percent and 27.8 percent, respectively. When the compression load was 1500N, the shear modulus and torsion strength decreased by 22.0 percent and 15.0 percent, respectively. The range of the average shear modulus was from 5.44±0.64GPa to 6.97±0.70GPa, and the range of the average torsion strength was from 47.92±7.80MPa to 66.31±7.51MPa. Generally, both the shear modulus and torsion strength all decreased with the increasing of the compression load. Because of the limited number and the non-uniform anisotropy of the bone materials, there were some slight fluctuations on the shear modulus and the torsion strength, when the compression load was 1000N and 1500N, respectively. However, in general, the mechanical properties of both shear modulus and torsion strength still decreased compared with the mechanical properties under the single torsion load. That could be seen that the torsion properties were degraded by the coupling loading of the compression load and the torsion load.

## Discussion

**Fig 4:**
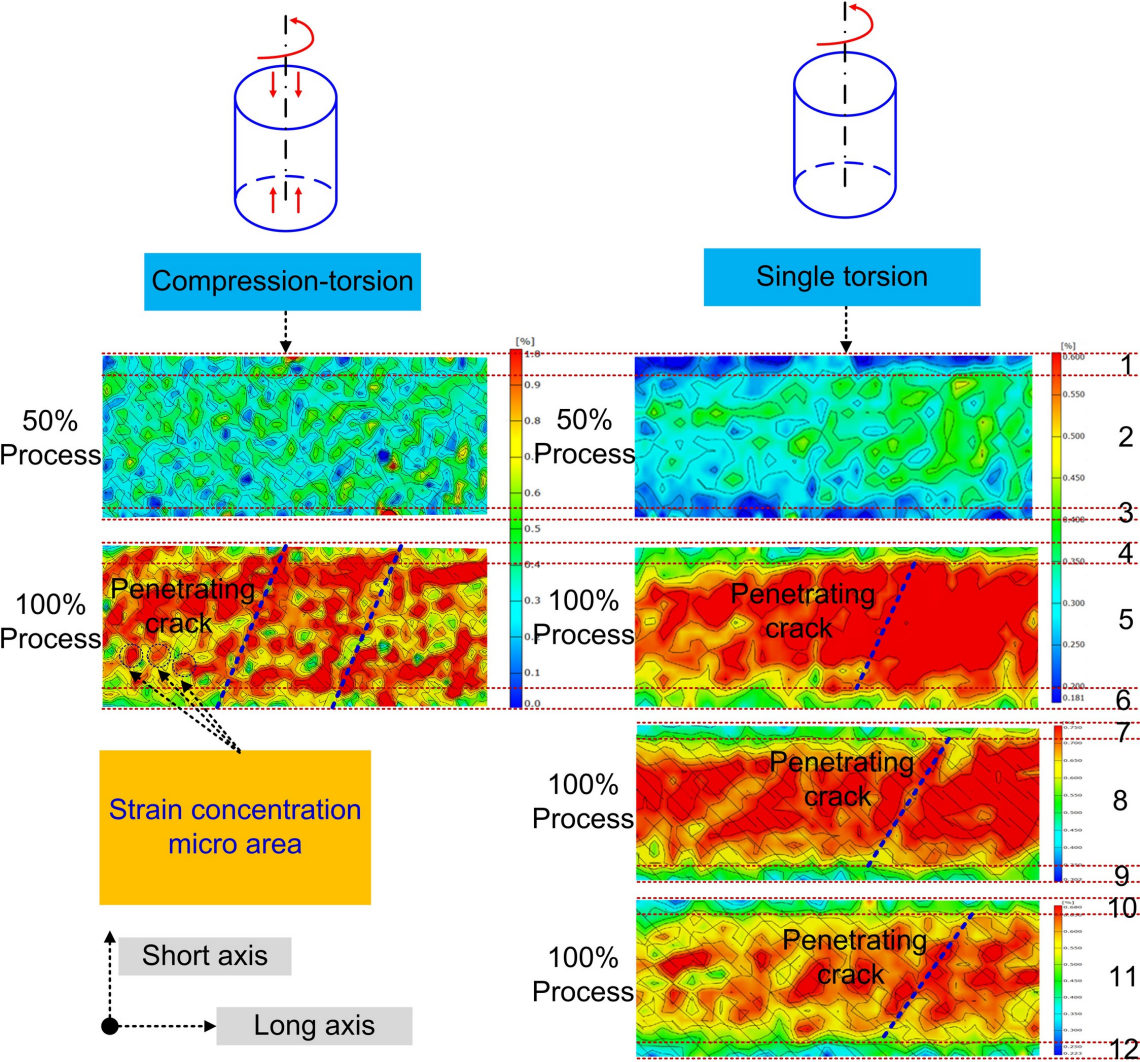
Principal strain contour of bone specimen under the compression-torsion coupling loads in different fracture stage (The distribution of the color of the strain contour was related to the spraying quality of the speckle, without affecting the revealing of the strain evolution mechanism. 1, 3, 4, 6, 7, 9, 10, and 12 are edge areas, 2, 5, 8 and 11 are the middle areas).

Although results indicated that the torsion mechanical properties weakened under the compression load, the potential mechanism remained unknown. As shown in Fig 4, the variations of the principal strain of the bone specimens were obtained by in-situ observation method in real-time. By image processing, the principal strain contours of bone specimens under both single torsion load and compression-torsion coupling loads in different fracture stage were obtained.

In Fig 4, from the evolution of the principal strain contour, the fracture process of the cortical bone could be summarized as follows. The short axis represented the transverse direction of the specimen, and long axis represented the longitudinal direction of the specimen. In the initial stage, the discrete principal strain concentration micro-area appeared. Then, the principal strain increased and the principal strain concentration micro-areas started to merge. Finally, the fracture was caused by the penetration of the principal strain concentration bands. However, compared to the single torsion’s non-penetrating cracks, it was found that the cracks under the compression-torsion coupling loads went across the short axis of the bone specimens. As shown in Fig 4, there was no slanting strain concentration band under the single torsion load in the areas of 1, 3, 4, 6, 7, 9, 10, and 12. However, the principal strain concentration bands went across all the short axis of the cortical bone specimens. At the same time, the principal strain concentration areas were uniformly distributed on the surface of the cortical bone specimens, rather than only being concentrated in the middle (areas 2, 5, 8 and 11) of the cortical bone specimens under the single torsion load. This suggested that the additional loading of the compression load changed the distribution of the surface principal strain. The principal strain concentration bands penetrated the short axis of the bone specimens, there were no restrictions to the crack propagation, so that this fracture was easier than fracture based on crack propagation in the internal spaces. Therefore, the shear modulus and torsion strength decreased.

On the other hand, from the uniform distribution of the principal strain, it could be seen that the anisotropy of bone specimen under the compression-torsion coupling loads was weakened sooner than that under single torsion load. As is well known, the excellent mechanical properties of bone materials depend on its natural anisotropy. Therefore, the rapid reduction of the anisotropy could promote the potential fracture probability in theory.

**Fig 5:**
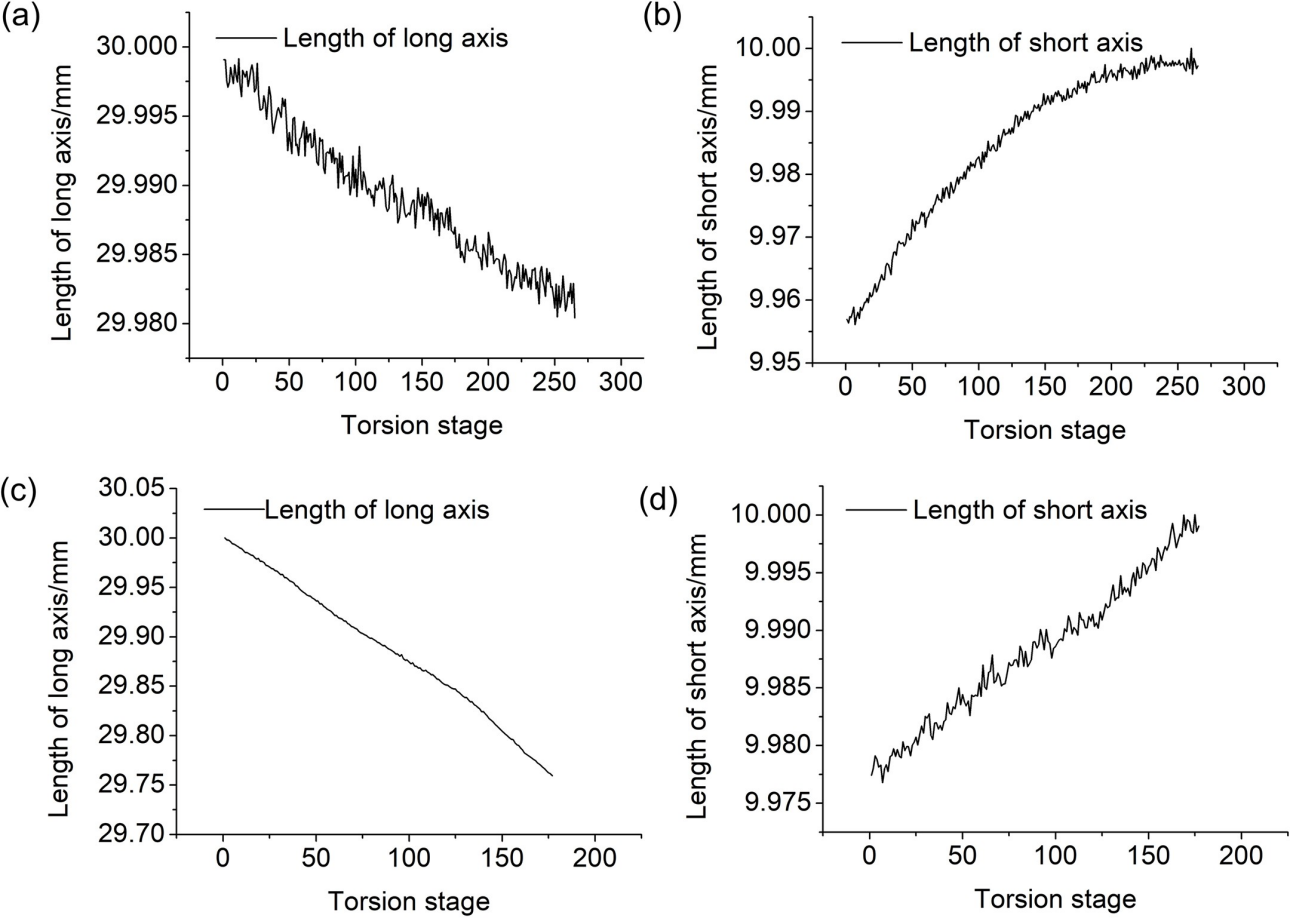
Variations of length of both long axis and short axis (a) Length of long axis under single torsion load with 0.3°/s torsion speed (b) Length of short axis under single torsion load with 0.3°/s torsion speed (c) Length of long axis under single compression load (d) Length of short axis under single compression load.

Generally speaking, the variations of mechanical properties were typically based on the deformation of the materials. Therefore, to investigating the potential influence mechanism of compression load on its torsion properties, the deformation of both long axis and short axis under both single torsion and single compression load was obtained, as shown in Fig 5. It could be seen that the length of long axis of specimens under both single torsion load and single compression load all decreased, and the length of short axis all increased. This suggested that the compression load could compensate for the deformation increment of the torsion load, and finally resulted in the accelerating of the torsion fracture. However, the fracture deformation component from the single torsion load decreased, so that the torsion strength and torsion modulus also decreased.

**Fig 6:**
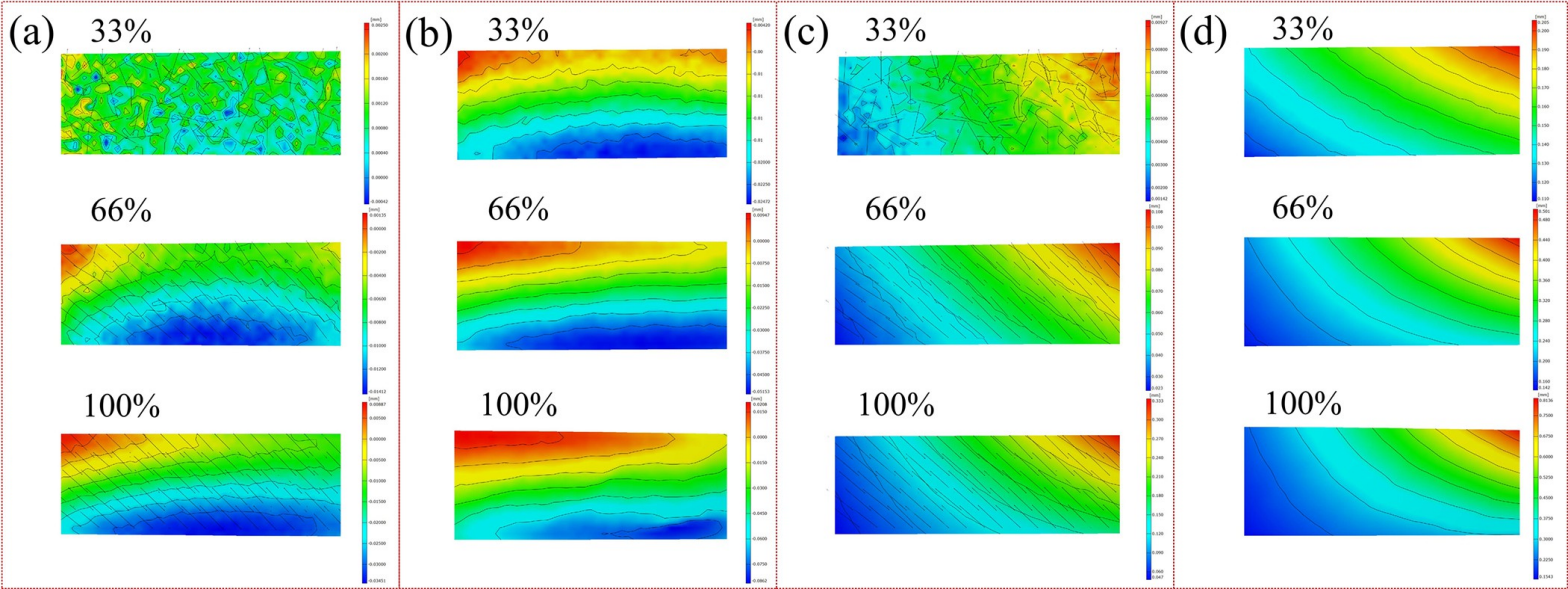
Strain contour of both long axis length and short axis length at different stages (a) Long axis of specimen under single torsion load (b) Long axis of specimen under compression-torsion coupling loads (c) Short axis of specimen under single torsion load (d) Short axis of specimen under compression-torsion coupling loads.

As shown in Fig 6, the variations of strain of both long axis length and short axis length under different loading patterns were obtained. From the evolution of the strain contour, it could be seen that the more regular distribution of strain was induced by compression load. This also suggested that the faster reduction of anisotropy was achieved with the adding of compression load. It was consistent with the analysis in Fig 4.

**Fig 7:**
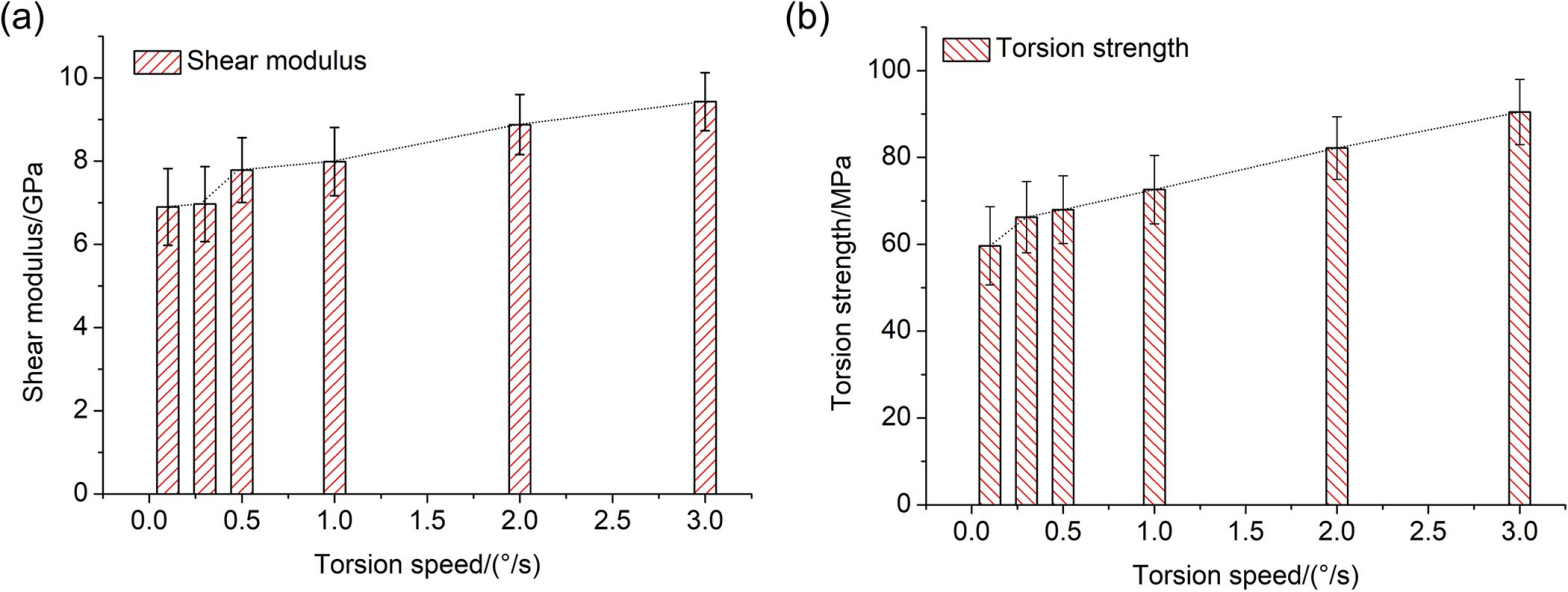
The shear modulus and torsion strength of bone specimen under single torsion load with different torsion speeds (a) Shear modulus (b) Torsion strength.

As analyzed above, the compression load promoted the effect of torsion properties. One part of the whole torsion speed was from compression load, and the other part was from the torsion load. Therefore, it meant that the pure torsion speed from torsion load was reduced under the compression load. That is to say, the torsion fracture should be easier under compression load, and the torsion speed component from single torsion load should decrease until fracture. This indicates that the bigger the compression load, the less the torsion speed, and the less the torsion properties. In order to provide further confirmation of the promotion effect of compression load on torsion load, the torsion properties under different torsion speeds were investigated, as shown in Fig 7. The results demonstrated that the shear modulus and the torsion strength all decreased with the decrease of the torsion speed. Therefore, the bigger compression load resulted in the less torsion speed, and also weakened the torsion properties.

**Fig 8:**
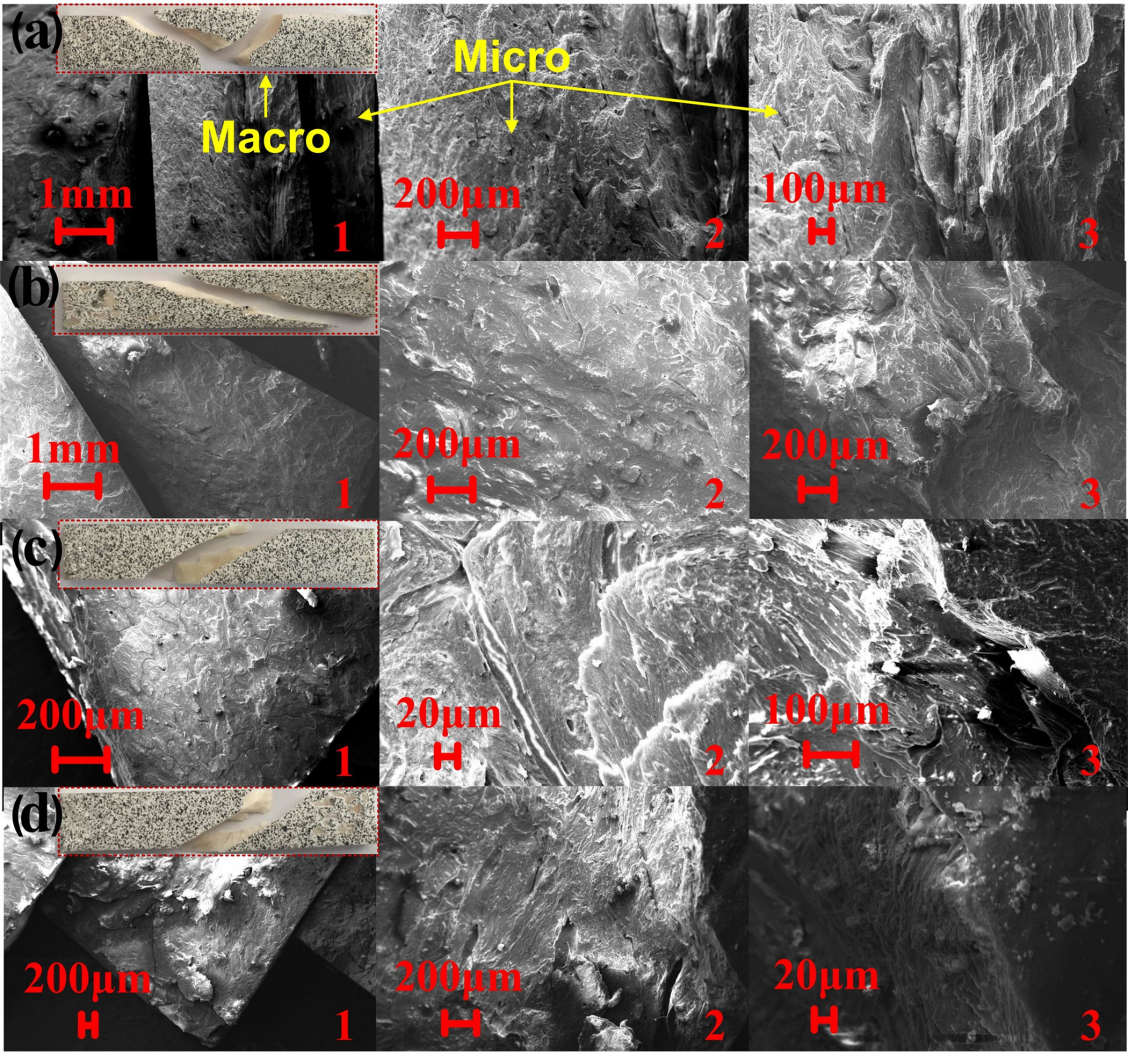
Macro and micro fracture morphology with different compression load (a) No compression, torsion speed 0.3°/s (b) 500N compression, torsion speed 0.3°/s (c) 1000N compression, torsion speed 0.3°/s (d) 1500N compression, torsion speed 0.3°/s.

Microstructure was known to have decisive effects on the mechanical properties of the bone materials [26–28]. For further investigation of the potential mechanism, the macro and micro fracture morphology were obtained by field-emission scanning electron microscope (FE-SEM), as shown in Fig 8.

Based on the directions and surface features of the fracture, it could be seen that the transverse fracture was the main feature under single torsion load. Under the compression-torsion coupling loads, there were both an obvious longitudinal fracture feature and an obvious transverse fracture feature, and the surface was rough. As was well known, as the microstructure of bone, the haversian system was orientated along the long axis of bone. It was also the sampling direction of the bone specimens. Therefore, the transverse fracture was achieved by the crack propagation through the lamellae layers of haversian system.

However, the cement line in cortical bone was prone to damage in the form of microcracks that reduced the overall material properties [29]. Therefore, unlike transverse fracture, the longitudinal fracture was caused by the crack propagation along the lamellae and cement, in the form of cleavage fracture, so that it was easier than the development of the crack of transverse fracture. Therefore, the transverse fracture needed more energy to achieve than longitudinal fracture. Therefore, it could be speculated that the variations of fracture mode and crack propagation mechanism weakened the torsion properties.

Based on research of Fatihhi, mechanical property of bone failure under uniaxial evaluation was found to be over-estimated, and it couldn’t closely simulate the loading conditions *in vivo* [24]. The over-estimated failure under uniaxial loading was in line with the decreased trends of the torsion properties under compression-torsion coupling loads in this research. The cortical bone material was the longitudinally-oriented material, consisting of a central vascular cavity, surrounded concentrically by lamellae [30]. The cement lines provided hyper-mineralized interfaces [31], and such structure provided convenient sites for micro-crack formation and propagation. Therefore, the micro-structure of cortical bone in longitudinally was a preferential low-energy micro-structural path for the crack propagation [32,33]. Koester found that the bone was far easier to split than to break along the long axis [34]. Fantner believed that the unidirectional cracks within fibers were the reason on single compression failures, and the micro-crack propagated initiated from the interface of the mineralized collagen fibers [35]. This cracks propagation mode explained why there was cleavage crack component on the total fracture under compression-torsion coupling loads. It was also completely consistent with the notion that the fracture of cortical bone was strongly associated with the path of the cracks in relation to the bone-matrix micro-structure. For the longitudinally-oriented materials, including cortical bone [38], wood [36,37], the fracture properties progressively decreased with increasing of the loading modes [38], which indicated the resistance to fracture extended from single direction to multi-direction [39,40]. Therefore, the fracture possibility increased based on decentralization of the cracks. Therefore, in line with previous studies, it demonstrated the cortical bone material was more dangerous under the complex loading patterns than under the single loading pattern. Therefore, to enhance the safety of cortical bone in service, reducing or avoiding the compression load component in torsion condition was the potential effective strategy.

## Conclusions

In this research, the mechanical response and deformation mechanism of cortical bone in multi-scale under the compression-torsion coupling loads were investigated by in-situ experiments. The conclusions are as follows.

1. The torsion strength and shear modulus under compression-torsion coupling loads were less than that under the single torsion load. The weakened effect increased with the increasing of compression load, within a limited extent. Mechanical evaluation under single loading pattern could result in an overestimate on the mechanical properties, indicating an inaccurate and dangerous judgement on the service conditions.
2. Based on in-situ observation, the comprehensive fracture patterns containing both transverse and longitudinal fractures were produced, with cracks propagated in transverse direction together with longitudinal direction, which resulted in the rapid reduction of the anisotropy of bone material.
3. The compression load could induce more fractures which were characterized by the cracks propagation along the low-energy interface of lamellae. By the variations of the cracks propagation and redistribution of the principal strain of the bone material, the torsion properties weakened under the compression load.
4. Based on this research, future works on bionic design and material optimization of bone tissue engineering materials used for bone repairing and bone substitute should take into consideration the crack arresting in the longitudinal direction.

This research would give some new understanding and theoretical references in area on objective service mechanical property of cortical bone material under complex patterns, and provide references for design of high performance bone substitutes with the functions of crack arrest and energy absorption.

## Supporting information

S1 Table The dimensions of the cortical bone specimen. (DOCX)

S2 Table The coefficient of the rectangle specimen under torsion load. (DOCX)
