# Supplementary material for: Mechanical response and in-situ deformation mechanism of cortical bone materials under combined compression and torsion loads
Source: PLoS One. 2022 Jul 27;17(7):e0271301. doi: 10.1371/journal.pone.0271301 (PMC9328520; doi:10.1371/journal.pone.0271301)
Supplement: S1 Table — (DOCX) [file pone.0271301.s001.docx]

**Table 1. The dimensions of the cortical bone specimen**

| **Material** | **Length(*h*)** | **Width(*b*)** | **Height(*l*)** |
| --- | --- | --- | --- |
| **Cortical bone** | 10.00mm | 3.00mm | 50.00mm |
