# Supplementary material for: Mechanical response and in-situ deformation mechanism of cortical bone materials under combined compression and torsion loads
Source: PLoS One. 2022 Jul 27;17(7):e0271301. doi: 10.1371/journal.pone.0271301 (PMC9328520; doi:10.1371/journal.pone.0271301)
Supplement: S2 Table — (DOCX) [file pone.0271301.s002.docx]

**Table 2. The coefficient of the rectangle specimen under torsion load**

| ***h/b*** | **1.0** | **1.2** | **1.5** | **2.0** | **2.5** | **3.0** | **4.0** | **6.0** | **8.0** | **10.0** | **∞** |
| --- | --- | --- | --- | --- | --- | --- | --- | --- | --- | --- | --- |
| ***α*** | 0.208 | 0.219 | 0.231 | 0.246 | 0.258 | 0.267 | 0.282 | 0.299 | 0.307 | 0.313 | 0.333 |
